# Robust and Adaptive Online Time Series Prediction with Long Short-Term Memory

**DOI:** 10.1155/2017/9478952

**Published:** 2017-12-17

**Authors:** Haimin Yang, Zhisong Pan, Qing Tao

**Affiliations:** ^1^College of Command and Information System, PLA University of Science and Technology, Nanjing, Jiangsu 210007, China; ^2^1st Department, Army Officer Academy of PLA, Hefei, Anhui 230031, China

## Abstract

Online time series prediction is the mainstream method in a wide range of fields, ranging from speech analysis and noise cancelation to stock market analysis. However, the data often contains many outliers with the increasing length of time series in real world. These outliers can mislead the learned model if treated as normal points in the process of prediction. To address this issue, in this paper, we propose a robust and adaptive online gradient learning method, RoAdam (Robust Adam), for long short-term memory (LSTM) to predict time series with outliers. This method tunes the learning rate of the stochastic gradient algorithm adaptively in the process of prediction, which reduces the adverse effect of outliers. It tracks the relative prediction error of the loss function with a weighted average through modifying Adam, a popular stochastic gradient method algorithm for training deep neural networks. In our algorithm, the large value of the relative prediction error corresponds to a small learning rate, and vice versa. The experiments on both synthetic data and real time series show that our method achieves better performance compared to the existing methods based on LSTM.

## 1. Introduction

A time series is a sequence of real-valued signals that are measured at successive time intervals ‎[1, 2]. Time series data occur naturally in many application areas such as economics, finance, environment, and medicine and often arrives in the form of streaming in many real-world systems. Time series prediction has been successfully used in a wide range of domains including speech analysis ‎[3], noise cancelation ‎[4], and stock market analysis ‎[5, 6]. The traditional methods of time series prediction commonly use a potential model, for example, autoregressive moving average (ARMA) ‎[7], autoregressive integrated moving average (ARIMA) ‎[1], and vector autoregressive moving average (VARMA) ‎[8], to mimic the data. However, these methods all need to deal with the whole dataset to identify the parameters of the model when facing new coming data, which is not suitable for large datasets and online time series prediction. To address this problem, online learning methods are explored to extract the underlying pattern representations from time series data in a sequential manner. Compared to traditional batch learning methods, online learning methods avoid expensive retraining cost when handling new coming data. Due to the efficiency and scalability, online learning methods including methods based on linear models ‎[9], ensemble learning ‎[10], and kernels ‎[11] have been applied to time series prediction successfully.

Long short-term memory (LSTM) ‎[12], a class of recurrent neural networks (RNNs) ‎[13], is particularly designed for sequential data. LSTM has shown promising results for time series prediction. Its units consist of three gates: input gate, forget gate, and output gate. It is popular due to the ability of learning hidden long-term sequential dependencies, which actually helps in learning the underlying representations of time series. However, the time series data in real world often contains some outliers more or less especially in cyberattacks, which are commonly shown as anomalies in time series data monitoring some measurements of network traffic. Those outliers mislead the learning method in extracting the true representations of time series and reduce the performance of prediction.

In this paper, we propose an efficient online gradient learning method, which we call RoAdam (Robust Adam) for LSTM to predict time series in the presence of outliers. The method modifies Adam (Adaptive Moment Estimation) ‎[14], a popular algorithm for training deep neural networks through tracking the relative prediction error of the loss function with a weighted average. Adam is based on standard stochastic gradient descent (SGD) method without considering the adverse effect of outliers. The learning rate of RoAdam is tuned adaptively according to the relative prediction error of the loss function. The large relative prediction error leads to a smaller effective learning rate. Likewise, a small error leads to a larger effective learning rate. The experiments show that our algorithm achieves the state-of-the-art performance of prediction.

The rest of this paper is organized as follows.  Section 2 reviews related work. In Section 3, we introduce some preliminaries.  Section 4 presents our algorithm in detail. In Section 5, we evaluate the performance of our proposed algorithm on both synthetic data and real time series. Finally, Section 6 concludes our work and discusses some future work.

## 2. Related Work

In time series, a data point is identified as an outlier if it is significantly different from the behavior of the major points. Outlier detection for time series data has been studied for decades. The main work focuses on modeling time series in the presence of outliers. In statistics, several parametric models have been proposed for time series prediction. The point that deviated from the predicted value by the summary parametric model including ARMA ‎[15], ARIMA ‎[16, 17], and VARMA ‎[18] is identified as an outlier. Vallis et al. ‎[19] develop a novel statistical technique using robust statistical metrics including median, median absolute deviation, and piecewise approximation of the underlying long-term trend to detect outliers accurately. There also exist many machine learning models for time series prediction with outliers. The paper ‎[20] proposes a generic and scalable framework for automated time series anomaly detection including two methods: plug-in method and decomposition-based method. The plug-in method applies a wide range of time series modeling and forecasting models to model the normal behavior of the time series. The decomposition-based method firstly decomposes a time series into three components: trend, seasonality, and noise and then captures the outliers through monitoring the noise component. The paper ‎[21] gives a detailed survey on outlier detection.

LSTM has shown promising results for time series prediction. Lipton et al. uses LSTM to model varying length sequences and capture long range dependencies. The model can effectively recognize patterns in multivariate time series of clinical measurements ‎[22]. Malhotra et al. use stacked LSTM networks for outliers detection in time series. A predictor is used to model the normal behavior and the resulting prediction errors are modeled as a multivariate Gaussian distribution, which is used to identify the abnormal behavior ‎[23]. Chauhan and Vig also utilize the probability distribution of the prediction errors from the LSTM models to indicate the abnormal and normal behaviors in ECG time series ‎[24]. These methods are not suitable for online time series prediction because they all need to train on time series without outliers to model the normal behavior in advance. In this paper, our online learning method for time series prediction is robust to outliers through adaptively tuning the learning rate of the stochastic gradient method to train LSTM.

## 3. Preliminaries and Model

In this section, we formulate our problem to be resolved and introduce some knowledge about Adam, a popular algorithm for training LSTM.

### 3.1. Online Time Series Prediction with LSTM

In the process of online time series prediction, the desirable model learns useful information from {*x*_1_, *x*_2_,…, *x*_*t*−1_} to give a prediction ${\tilde{x}}_{t}$ and then compare ${\tilde{x}}_{t}$ with *x*_*t*_ to update itself, where {*x*_1_, *x*_2_,…, *x*_*t*−1_} is a time series, ${\tilde{x}}_{t}$ is the time series data point forecasted at time *t*, and *x*_*t*_ is the real value. LSTM is suitable for discovering dependence relationships between the time series data by using specialized gating and memory mechanisms.

We give the formal definition of a neuron of a LSTM layer as follows. The* j*th neuron of a LSTM layer at time* t*, *c*_*t*_^*j*^consists of input gate *i*_*t*_^*j*^, forget gate *f*_*t*_^*j*^, and output gate *o*_*t*_^*j*^ and is updated through forgetting the partially existing memory and adding a new memory content ${\tilde{c}}_{t}^{j}$. The expressions of *i*_*t*_^*j*^, *f*_*t*_^*j*^, *o*_*t*_^*j*^, and *c*_*t*_^*j*^ are shown as follows:

(1) itj=σWixt+Uiht−1+Vict−1j,ftj=σWfxt+Ufht−1+Vfct−1j,otj=σWoxt+Uoht−1+Voct−1j,ctj=ftjct−1j+itjc~tj.

Note that *W*_*i*_, *W*_*f*_, *W*_*o*_, *U*_*i*_, *U*_*f*_, and *U*_*o*_ are the parameters of the *j*th neuron of a LSTM layer at time* t*. *σ* is a logistic sigmoid function. *V*_*i*_, *V*_*f*_, and *V*_*o*_ are diagonal matrices. *h*_*t*−1_ and *c*_*t*−1_ are the vectorization of *h*_*t*−1_^*j*^ and *c*_*t*−1_^*j*^. The output *h*_*t*_^*j*^ of this neuron at time *t* is expressed as

(2) $$\begin{array}{c} h_{t}^{j}=o_{t}^{j}\tanh\left(\;c_{t}^{j}\;\right). \end{array}$$

In our model of online time series prediction, we set a dense layer to map the outputs to the target prediction, which is formulated as

(3) $$\begin{array}{c} y=g\left(\;W_{d}h_{t}+b_{d}\;\right), \end{array}$$

where *g*(·) is the activation function of the dense layer, *W*_*d*_ is the weights, *b*_*d*_ is the bias, and *h*_*t*_ is the vectorization of *h*_*t*_^*j*^. The objection of our model at time *t* is to update the parameters *W*_*t*_ = {*W*_*i*_, *W*_*f*_, *W*_*o*_, *W*_*d*_, *U*_*i*_, *U*_*f*_, *U*_*o*_, *V*_*i*_, *V*_*f*_, *V*_*o*_, *b*_*d*_}. The standard process is

(4) $$\begin{array}{c} W_{t+1}=W_{t}-\eta \nabla l\left(\;x_{t},{\tilde{x}}_{t}\;\right), \end{array}$$

where *η* is the learning rate and $l(x_{t},{\tilde{x}}_{t})$ is the loss function.

### 3.2. Adam

Adam is a method for efficient stochastic optimization, which is often used to train LSTM. It computes adaptive learning rates for individual parameters from estimates of the first moment and the second moment of the gradients, only requiring first-order gradients. Adam keeps an exponentially decaying average of the gradient and the squared gradient:

(5) mt=β1mt−1+1−β1gt,vt=β2vt−1+1−β2gt2,

where *m*_*t*_ and *v*_*t*_ initialized as zero are estimates of the first moment and the second moment and *β*_1_ and *β*_2_ are exponential decay rates for the moment estimates. We can find that *m*_*t*_ and *v*_*t*_ are biased towards zero, when *β*_1_ and *β*_2_ are close to 1. So Adam counteracts these biases through bias correction of *m*_*t*_ and *v*_*t*_:

(6) m^t=mt1−β1t,v^t=vt1−β2t.

The rule of updating parameters is

(7) $$\begin{array}{c} W_{t+1}=W_{t}-\frac{\eta }{\sqrt{{\hat{v}}_{t}}+\epsilon }{\hat{m}}_{t}, \end{array}$$

where *η* = 0.001, *β*_1_ = 0.9, *β*_2_ = 0.999, and *ϵ* = 10^−8^ by default.

## 4. Method

In this section, we introduce our online gradient learning method, which is called RoAdam (Robust Adam) to train long short-term memory (LSTM) for time series prediction in the presence of outliers. Our method does not directly detect the outliers and adaptively tunes the learning rate when facing a suspicious outlier.

In Algorithm 1, we provide the details of the RoAdam algorithm. The main difference between our algorithm and Adam is *r*_*t*_, a relative prediction error term of the loss function. The relative prediction error term indicates whether the point is an outlier. The larger value of *r*_*t*_ means the current point is more suspicious to be an outlier. It is computed as *r*_*t*_ = ‖*l*(*W*_*t*−1_)/*l*(*W*_*t*−2_)‖, where $l\left(W_{t-1}\right)=l(x_{t},{\tilde{x}}_{t})$ and $l\left(W_{t-2}\right)=l\left(x_{t-1},{\tilde{x}}_{t-1}\right)$. $l\left(x_{t},{\tilde{x}}_{t}\right)$ and $l\left(x_{t-1},{\tilde{x}}_{t-1}\right)$ are the absolute prediction errors of *x*_*t*_ and *x*_*t*−1_. In practice, a threshold is used to scheme to ensure the stability of relative prediction error term. *k* and *K* denote the lower and upper thresholds for *r*_*t*_. We let *r*_*t*_ = min⁡{max⁡{*k*, ‖*l*(*W*_*t*−1_)/*l*(*W*_*t*−2_)‖}, *K*} (1), if ‖*l*(*W*_*t*−1_)‖ ≥ ‖*l*(*W*_*t*−2_)‖ and *r*_*t*_ = min⁡{max⁡{1/*K*, ‖*l*(*W*_*t*−1_)/*l*(*W*_*t*−2_)‖}, 1/*k*} (2) otherwise, which captures both increase and decrease of relative prediction error. Our settings consider different situations when the preceding point *x*_*t*−1_ and current point *x*_*t*_ are at different status. The details are listed in Table 1.

To get a smoother estimate, we compute the relative prediction error with a weighted average. The final result *d*_*t*_ is *β*_3_*d*_*t*−1_ + (1 − *β*_3_)*r*_*t*_. Here the effect of *β*_3_ is the same as *β*_1_ and *β*_2_ in Adam. In general, RoAdam is modified in the basis of Adam through multiplying the denominator $\sqrt{{\hat{v}}_{t}}$ with *d*_*t*_. The large value of *d*_*t*_ corresponds to a small learning rate, and vice versa.

## 5. Experiment

In this section, we illustrate the performance of our proposed algorithm RoAdam compared to RLSTM, SR-LSTM, and RN-LSTM on both synthetic data and real time series.

### 5.1. Experiment Setup

RLSTM means real time LSTM, which updates the model using the newly coming data without considering the effect of outliers. SR-LSTM stands for LSTM with suspicious point removal. The difference between SR-LSTM and RN-LSTM is that once a suspicious point is detected as an outlier, SR-LSTM does not update on this point and RN-LSTM updates using a recent normal point. They both use the method proposed in ‎[25] to detect the outlier. In addition, all the algorithms use the same LSTM model besides the optimizer. RLSTM, SR-LSTM, and RN-LSTM adopt the original Adam optimizer. The LSTM model has 3 layers and the number of neurons in each layer is 400. The mean squared error is chosen as the loss function and the L2 regularization with 0.0001 penalty is used. The parameters of RoAdam carried from Adam have the same default values: *η* = 0.001, *β*_1_ = 0.9, *β*_2_ = 0.999, and *ϵ* = 10^−8^. For parameters specific to our method, we try different values and recommend default values *β*_3_ = 0.999, *k* = 0.1, and *K* = 10.

### 5.2. Data Sets

To examine the prediction performance, we evaluate all the previous algorithms on synthetic data and real time series.

#### 5.2.1. Synthetic Data

The synthetic data is sampled from a Gaussian distribution with the corresponding mean *u* ∈ [0,100] and variance *σ* ∈ [10,30] plus the trend component *T* ∈ [−0.5,0.5]. The length *l* is 2,500. The outliers are injected based on a Bernoulli distribution identified by *α* = 0.01 and *l* · *α* is the expected number of outliers. The values of outliers are also sampled from a Gaussian distribution with mean *u* ∈ [0,1000] and variance *σ* ∈ [10,30]. The expression of *x*_*t*_ is

(8) $$\begin{array}{c} x_{t}=\left\{\begin{array}{cc} x+T,x\sim N\left(u,\sigma \right),u\in \left[0,100\right],\sigma \in \left[10,30\right],T\in \left[-0.5,0.5\right], & \text{when}\;\;x_{t}\text{ is a normal point};\\ x,x\sim N\left(u,\sigma \right),u\in \left[0,1000\right],\sigma \in \left[10,30\right], & \text{when }x_{t}\text{ is an outlier}. \end{array}\right. \end{array}$$

#### 5.2.2. Real Time Series

**Figure 1:**
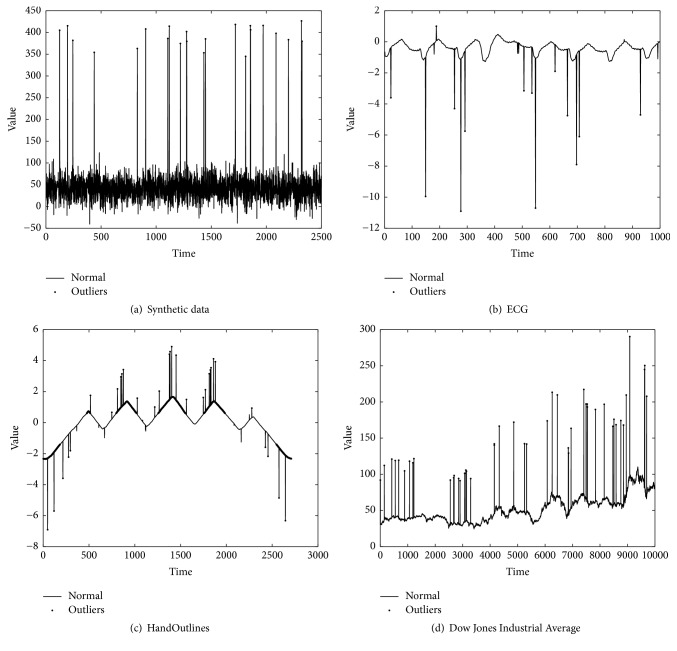
True value of data sets.

The first time series data is ECG data, which consists of 70 series of 1000 ECG measurements ‎[26]. We choose 100 samples from ECG data set. The second one is HandOutlines, which is from the commonly used UCR (http://www.cs.ucr.edu/~eamonn/time_series_data/.). The last time series data is daily index of Dow Jones Industrial Average (DJIA) during years 1885–1962. We randomly select 1% of each real time series as outliers, whose values are 2 or 3 times bigger than the true ones.  Figure 1 presents the true value of synthetic data and real time series. The *x*-axis is time (the number of samples) and the *y*-axis is true value.

### 5.3. Experimental Results

In this section, we test RMSE of the algorithms mentioned above to examine the effectiveness and efficiency.

(9) $$\begin{array}{c} RMSE=\frac{1}{T}\overset{T}{\underset{t=1}{\sum }}{\left(\;X_{t}-{\tilde{X}}_{t}\;\right)}^{2}. \end{array}$$

RMSE allows us to compare errors with the number of samples increasing. In addition, we average the results over 100 runs for stability.

**Figure 2:**
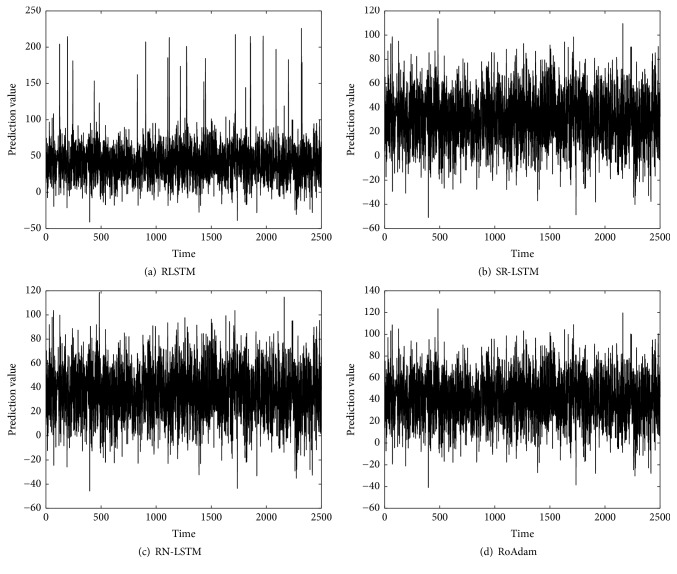
Prediction value of different algorithms on synthetic data.

**Figure 3:**
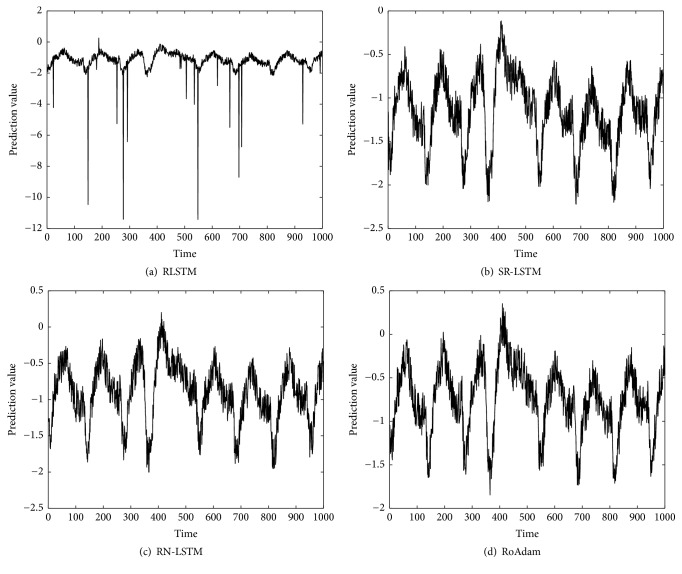
Prediction value of different algorithms on ECG.

**Figure 4:**
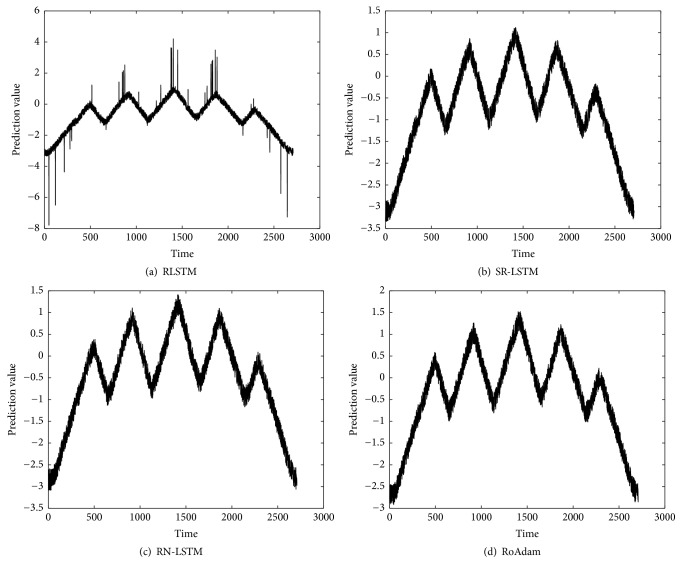
Prediction value of different algorithms on HandOutlines.

**Figure 5:**
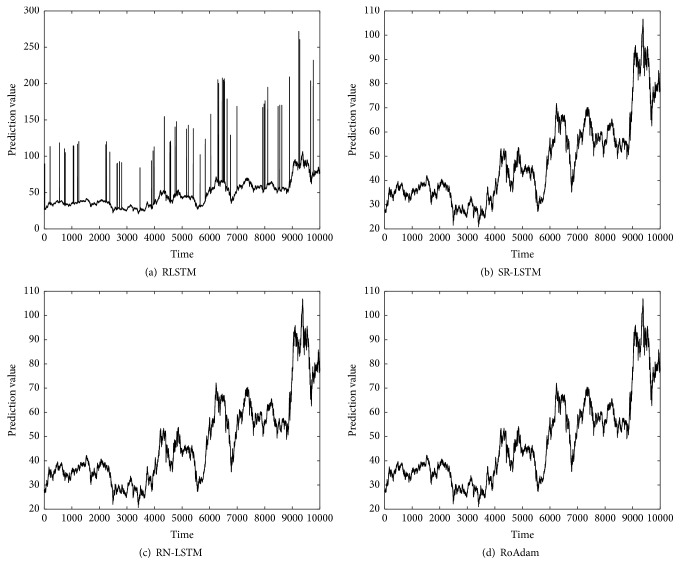
Prediction value of different algorithms on DJIA.

Table 2 shows the RMSE of different algorithms both on synthetic data and real time series. We can find that RoAdam outperforms all the other algorithms on RMSE. Figures 2–5 visualize the prediction value of all the algorithms on synthetic data and real time series. The *x*-axis is time (the number of samples) and the *y*-axis is prediction value. We can observe that the prediction value produced by RLSTM has oscillations around outliers. It indicates that the prediction performance of RLSTM is indeed affected by outliers. Although SR-LSTM, RN-LSTM, and RoAdam have almost the same shape of prediction value, RoAdam has the least RMSE. The reason may be that SR-LSTM and RN-LSTM may lose some information of the normal points when they are mistaken outliers.

## 6. Conclusions

In this paper, we propose an efficient online gradient learning method, RoAdam for LSTM, to predict time series, which is robust to outliers. RoAdam is modified on the basis of Adam, a popular stochastic gradient algorithm for training deep neural networks. Through tracking the relative prediction error of the loss function with a weighted average, this method adaptively tunes the learning rate of the stochastic gradient method in the presence of outliers. In the process of prediction, the large value of the relative prediction error corresponds to a small learning rate, and vice versa. The experiments on both synthetic data and real time series show that our method achieves less prediction error compared to the existing methods based on LSTM.

It remains for future work to study whether our approach could be extended to time series prediction with missing data.

## Figures and Tables

**Algorithm 1:**
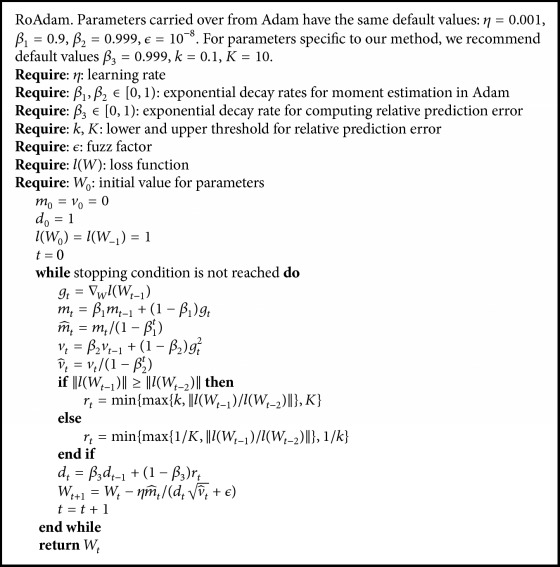


**Table 1:** Different values of *r*_*t*_.

*x*_*t*−1_	*x*_*t*_	
	Outlier	Normal
Outlier	(1)	(2)
Normal	(2)	(1)

**Table 2:** RMSE on synthetic data and real time series.

Algorithm	Data			
	Synthetic	ECG	HandOutlines	DJIA
RLSTM	0.7606	0.8505	0.9756	1.8454
SR-LSTM	0.7329	0.8323	0.9411	1.7574
RN-LSTM	0.7218	0.8217	0.9376	1.6218
RoAdam	**0.4946**	**0.5626**	**0.7633**	**1.3875**

## References

[1] Hamilton J. D. (1994). *Time Series Analysis*.

[2] Brockwell P. J., Davis R. A. (2006). *Time Series: Theory and Methods*.

[3] Rabiner L. R., Schafer R. W. (1978). *Digital processing of speech signals*.

[4] Gao J., Sultan H., Hu J., Tung W.-W. (2010). Denoising nonlinear time series by adaptive filtering and wavelet shrinkage: a comparison. *IEEE Signal Processing Letters*.

[5] Granger C. W. J., Newbold P. (1986). *Forecasting Economic Time Series*.

[6] Nerlove M., Grether D. M., Carvalho J. L. (1979). *Analysis of Economic Time Series: A Synthesis*.

[7] Rojo-Alvarez J. L., Martınez-Ramon M., de Prado-Cumplido M. (2004). Support vector method for robust ARMA system identification. *IEEE Transactions on Signal Processing*.

[8] Tsay R. S. (2014). *Multivariate Time Series Analysis: with R And Financial Applications*.

[9] Anava O., Hazan E., Mannor S. (2013). Online learning for time series prediction. *Journal of Machine Learning Research*.

[10] Minku L. L., Yao X. (2012). DDD: a new ensemble approach for dealing with concept drift. *IEEE Transactions on Knowledge and Data Engineering*.

[11] Richard C., Bermudez J. C., Honeine P. (2008). Online prediction of time serise data with kernels. *IEEE Transactions on Signal Processing*.

[12] Hochreiter S., Schmidhuber J. (1997). Long short-term memory. *Neural Computation*.

[13] LeCun Y., Bengio Y., Hinton G. (2015). Deep learning. *Nature*.

[14] Kingma D. P., Ba J. L. Adam: a method for stochastic optimization.

[15] Barnett V., Lewis T. (1978). *Outliers in Statistical Data*.

[16] Hawkins D. M. (1980). *Identification of Outliers*.

[17] Rousseeuw P. J., Leroy A. M. (1987). *Robust Regression and Outlier Detection*.

[18] Tsay R. S. (1986). Time series model specification in the presence of outliers. *Journal of the American Statistical Association*.

[19] Vallis O., Hochenbaum J., Kejariwal A. A novel technique for long-term anomaly detection in the cloud.

[20] Laptev N., Amizadeh S., Flint I. Generic and scalable framework for automated time-series anomaly detection.

[21] Gupta M., Gao J., Aggarwal C. C., Han J. (2014). Outlier detection for temporal data: a survey. *IEEE Transactions on Knowledge and Data Engineering*.

[22] Lipton Z. C., Kale D. C., Elkan C. https://arxiv.org/pdf/1511.03677.pdf.

[23] Malhotra P., Vig L., Shroff G., Agarwal P. Long Short Term Memory networks for anomaly detection in time series.

[24] Chauhan S., Vig L. Anomaly detection in ECG time signals via deep long short-term memory networks.

[25] Connor J. T., Martin R. D., Atlas L. E. (1994). Recurrent neural networks and robust time series prediction. *IEEE Transactions on Neural Networks and Learning Systems*.

[26] Bagnall A. J., Janacek G. J. Clustering time series from ARMA models with clipped data.

